# LPIN3 emerges as a diagnostic biomarker in Moyamoya disease revealing immune-lipid metabolic crosstalk

**DOI:** 10.3389/fgene.2026.1853818

**Published:** 2026-06-09

**Authors:** Zhenwei Lu, Xiansheng Qiu, Liwei Zhou, Xiaodong Li, Hanwen Lu, Shuo Wang, Junfu Chen, Lifei Bian, Jianbin Lin, Wenpeng Zhao, Wujie Zhao, Xin Gao, Jinsen Zhang, Sifang Chen, Zhangyu Li, Zhanxiang Wang

**Affiliations:** 1 The Graduate School of Fujian Medical University, Fuzhou, China; 2 Department of Neurosurgery and Department of Neuroscience, The First Affiliated Hospital of Xiamen University, School of Medicine, Xiamen University, Xiamen, Fujian, China; 3 Fujian Key Laboratory of Brain Tumors Diagnosis and Precision Treatment, Xiamen Key Laboratory of Brain Center, The First Affiliated Hospital of Xiamen University, Xiamen, Fujian, China; 4 Fujian University of Traditional Chinese Medicine, Fuzhou, China; 5 School of Life Science, Xiamen University, Xiamen, China; 6 School of Medicine, Xiamen University, Xiamen, Fujian, China; 7 Department of Pain Medicine, The First Affiliated Hospital of Xiamen University, Xiamen, Fujian, China

**Keywords:** immune infiltration, lipid metabolism, machine learning, Moyamoya disease, WGCNA

## Abstract

**Background:**

Moyamoya disease (MMD) is a progressive cerebrovascular disorder characterized by stenosis or occlusion of the terminal portions of the internal carotid arteries and their proximal branches, accompanied by the formation of abnormal collateral vessel networks. It represents a leading cause of ischemic and hemorrhagic stroke in both pediatric and adult populations. However, a comprehensive understanding of the molecular drivers underlying the hallmark vascular pathology of MMD remains elusive. Emerging evidence indicates that dysregulated lipid metabolism significantly contributes to MMD susceptibility and disease severity; nevertheless, its precise mechanistic roles in MMD pathogenesis have not been thoroughly investigated.

**Methods:**

We integrated three publicly available gene expression datasets comprising MMD patients and non-MMD controls (GSE189993, GSE157628, and GSE141024). Following rigorous batch-effect correction, differential expression analysis was performed to identify differentially expressed genes (DEGs). Gene set enrichment analysis (GSEA), weighted gene co-expression network analysis (WGCNA), and machine learning approaches were then integrated to prioritize hub genes. Immune cell infiltration analysis was conducted for the identified hub genes. Subsequently, functional enrichment analysis, immune infiltration profiling, and protein-protein interaction (PPI) network construction were further performed. Validation was carried out using an independent external dataset (GSE249254) as well as *in vitro* experiments-including hypoxia-treated human endothelial cells and patient-derived tissue samples-to assess mRNA and protein expression levels. Finally, a Transcription Factor (TF)-miRNA-mRNA regulatory network was constructed, and potential therapeutic compounds targeting MMD were predicted via computational screening.

**Results:**

A total of 2,288 DEGs were identified. GSEA revealed significant enrichment of pathways related to lipid metabolism and immune responses. WGCNA identified MMD-associated co-expression modules, and integrative machine learning prioritized four hub genes: LPIN3, PPT2, ACSS1, and INPPL1. A diagnostic nomogram built upon these four genes demonstrated robust predictive performance, with an area under the curve (AUC) of 0.91. Immune infiltration analysis revealed that the abundance of B cells in the MMD patient group was significantly lower than that in the control group, with statistical significance. Notably, LPIN3 expression was significantly upregulated in MMD. It was the only hub gene whose upregulation at the mRNA level was consistently validated in both the external validation set (GSE249254) and *in vitro* models. Subsequent immunohistochemical (IHC) experiments further corroborated this finding at the protein level, highlighting its potential as an independent biomarker. Furthermore, leveraging the hub gene network, seven candidate compounds with potential therapeutic relevance to MMD were predicted.

**Conclusion:**

This study delineates the immune-lipid metabolic transcriptomic characteristics of MMD, identifies novel molecular determinants of disease pathogenesis, and validates LPIN3 as a promising diagnostic biomarker. Collectively, these findings provide critical mechanistic insights into MMD etiology and offer a foundation for developing improved diagnostic strategies and targeted therapeutic interventions.

## 1 Introduction

Moyamoya disease (MMD) is a rare cerebrovascular disorder characterized by progressive stenosis of the terminal portions of the internal carotid arteries and their proximal branches, accompanied by the development of a fragile collateral vascular network (Scott and Smith, 2009; Shang et al., 2020). Although MMD is globally uncommon, its incidence is markedly higher in East Asian populations-particularly in Japan, Korea, and China (Mertens et al., 2022; Zhang et al., 2022)—and it represents a leading cause of stroke in both pediatric and adult patients. Surgical revascularization—most commonly superficial temporal artery-middle cerebral artery (STA-MCA) bypass—is the primary therapeutic intervention (Chen et al., 2023; Acker et al., 2018), aimed at restoring cerebral perfusion. However, this approach does not halt the underlying pathological progression of the disease. Thus, elucidating the fundamental molecular mechanisms of MMD remains a critical unmet need.

Despite decades of clinical observation and research, the precise etiology and pathogenesis of MMD remain largely elusive. Genetic studies have identified RNF213, also known as mysterin, as a susceptibility gene for MMD (Asselman et al., 2022; Fujimura et al., 2014). However, its incomplete penetrance and variable allele frequency across populations suggest that additional genetic modifiers and environmental triggers likely contribute to disease onset. Moreover, accumulating evidence implicates immune-mediated responses and vascular inflammation in the pathogenesis of MMD (Chen et al., 2023; He et al., 2025a). Histopathological analyses of affected intracranial arteries reveal immune cell infiltration-including macrophages and T lymphocytes—as well as aberrant expression of immune-related molecules within the thickened intima. Transcriptomic profiling of peripheral blood and vascular tissues further supports a pathogenic role for dysregulated immune responses in MMD progression (Zhang et al., 2019; Ihara et al., 2022). Nevertheless, the integrated mechanisms by which genetic predisposition, immune activation, and disease-specific transcriptional programs interact to drive the characteristic cerebrovascular pathology of MMD remain elusive.

Over the past two decades, advances in high-throughput omics technologies have provided powerful tools for deciphering the molecular underpinnings of complex diseases. Transcriptomic analyses in MMD have revealed dysregulation of immune-inflammatory responses, aberrant vascular remodeling, and metabolic pathways, all implicated in pathogenesis (Asselman et al., 2022; Liu et al., 2025; Ge et al., 2024; He et al., 2025b). Notably, disturbances in lipid and broader metabolic pathways have garnered increasing interest. Using an untargeted metabolomics approach that integrates ultra-high-performance liquid chromatography with high-resolution mass spectrometry, He et al. demonstrated a significant correlation between diacylglycerol (DAG) and triglyceride levels in intracranial arterial tissues from MMD patients (He et al., 2024), suggesting that DAG-mediated signaling dysregulation may play a role in MMD progression. Another prospective case-control study comprehensively profiled the lipidome and identified oxidized low-density lipoprotein (oxLDL) as a key biomarker (Zeng et al., 2025), further underscoring the critical involvement of lipid metabolism in MMD pathogenesis. Nevertheless, integrative molecular network studies linking immune infiltration features-such as alterations in T-cell and B-cell populations-to specific metabolic pathways-including fatty acid digestion and absorption-remain scarce. A more comprehensive and systematic investigation is therefore needed to elucidate how transcriptomic signatures relate to the dysregulated immune landscape within the MMD vascular microenvironment.

This study integrated bioinformatics analyses—including weighted gene co-expression network analysis (WGCNA), machine learning, and immune infiltration profiling—to identify lipid metabolism-associated signature genes in MMD. Based on these genes, a high-performance diagnostic model for MMD was constructed. Furthermore, a regulatory network was established and potential therapeutic agents were predicted. To ensure robustness, the identified signature genes were validated using independent external datasets. Finally, the expression and protein-level localization of the key gene were experimentally confirmed via quantitative real-time polymerase chain reaction (qRT-PCR) and immunohistochemistry (IHC).

## 2 Materials and methods

### 2.1 Downloaded data

**FIGURE 1:**
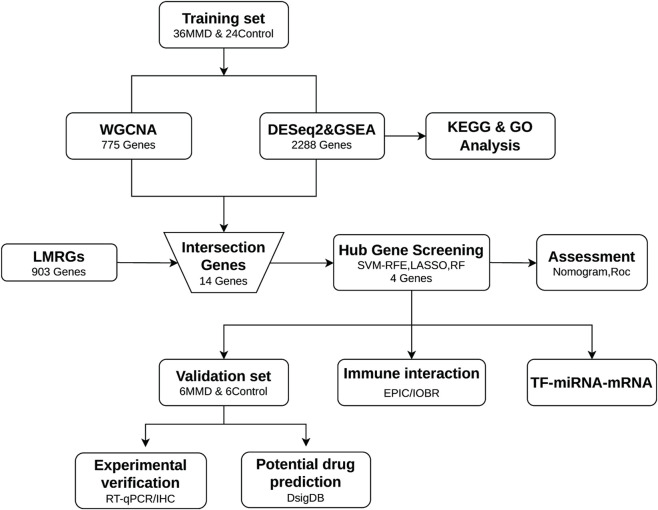
The flow chart of this study.

We searched the Gene Expression Omnibus (GEO) database using the keyword “Moyamoya disease” to identify and select relevant datasets. After removing duplicate subsets, we downloaded the raw microarray data from GSE189993, GSE157628, and GSE141024, as well as the RNA-seq data from GSE249254 for downstream analysis. The microarray platform used was the Agilent SurePrint G3 Human GE v2 8 × 60 K microarray, corresponding to GEO platform accession numbers GPL16699. The datasets GSE189993, GSE157628, and GSE141024 were pooled to constitute the training set, whereas GSE249254 served as the independent validation set. The training set comprised 36 patients with MMD and 24 non-MMD controls; the independent validation set comprised 6 patients with MMD and 6 non-MMD controls. Detailed characteristics of these datasets are summarized in Supplementary Table S1. The workflow of this study is illustrated in Figure 1.

We searched the Molecular Signatures Database (MSigDB; https://www.gsea-msigdb.org/gsea/msigdb) using the keyword “lipid metabolism” to retrieve lipid metabolism-related gene sets from the Hallmark, KEGG, Reactom, and WikiPathway collections. This search yielded six lipid metabolism-associated pathway gene sets (Supplementary Table S1). Following retrieval, these gene sets were merged, and duplicate genes were removed, resulting in a final compiled list of 903 lipid metabolism-related genes (LMRGs) for analysis (Supplementary Table S2).

### 2.2 Identification of differentially expressed genes (DEGs) and gene set enrichment analysis (GSEA)

**FIGURE 2:**
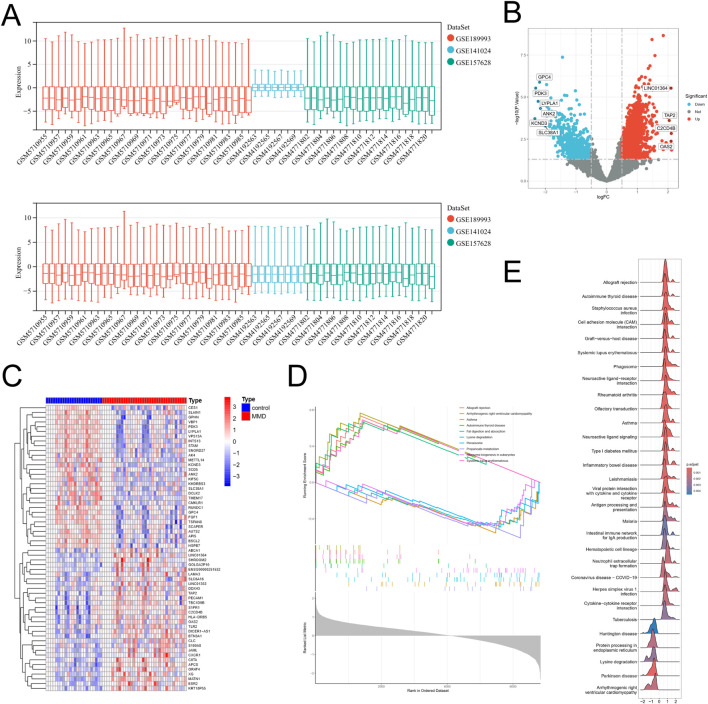
Data preprocessing and differential analysis. **(A)** Box plots of samples before and after batch correction. **(B)** Volcano plot of differentially expressed genes. Red dots indicate up-regulated genes, blue dots indicate downregulated genes. The horizontal black dotted line corresponds to the p-value threshold of 0.05, and the vertical black dotted line corresponds to |log_2_FC| = 0.5. **(C)** Cluster heatmap of expression levels of MMD-related DEGs. **(D)** GSEA analysis of DEGs. **(E)** Ridgeline plot of DEGs.

For the integration of multiple datasets, we first employed the R package “inSilicoMerging” to merge the datasets (Zhou et al., 2025). Subsequently, batch-effect correction and normalization were performed applying the “RemoveBatchEffect” function (Johnson et al., 2007), yielding a batch-corrected expression matrix. Differential expression analysis was then conducted using the “limma” R package (Ritchie et al., 2015). Genes were considered differentially expressed if they satisfied both criteria: |log_2_ (fold change)| > 0.5 and p-value <0.05. The results of the differential expression analysis were visualized using the R packages “ggplot2” and “pheatmap”: “ggplot2” was used to generate volcano plots, while “pheatmap” was employed to construct heatmaps depicting the expression patterns of DEGs (Figure 2).

Gene Set Enrichment Analysis (GSEA) was performed using the “GSEABase” R package to compute enrichment scores for predefined gene sets in each sample (Mootha et al., 2003). Gene sets were ranked based on their correlation with the MMD phenotype. Sets with a nominal p-value <0.05 were considered significantly enriched. Functional enrichment analysis of phenotype-associated genes was further conducted using the “clusterProfiler” R package, excluding gene sets containing fewer than 10 or more than 200 genes.

### 2.3 WGCNA

We performed WGCNA using the merged transcriptome matrix derived from the entire training set. The R package “WGCNA” was employed to construct the co-expression network. Specifically, the adjacency matrix was transformed into a topological overlap matrix (TOM); genes with a normalized expression value >0.5 were retained for downstream analysis. A soft-thresholding power (β) of 4 was selected to ensure scale-free topology, achieving a model fit index (R^2^) > 0.9. Modules were identified with a minimum size of 50 genes, and the dynamic tree-cutting height threshold was set to 0.25.

A module-trait association heatmap was generated to identify key modules significantly correlated with the clinical phenotype (MMD vs. control). Module-trait relationships were quantified by correlating module eigengenes with clinical traits, enabling robust identification of modules highly associated with phenotypic variation (Figure 7C). Gene significance (GS) for the binary trait (MMD vs. control) was calculated as −log_10_(p), where p is the p-value from the logistic regression model of individual gene expression against the trait. Module membership (MM) was computed as the correlation between each gene’s expression profile and the corresponding module eigengene. The module exhibiting the highest absolute correlation with the trait of interest was designated the key module and subjected to subsequent functional and biological interpretation.

### 2.4 Screening hub genes by machine learning and ROC curve analysis

**FIGURE 3:**
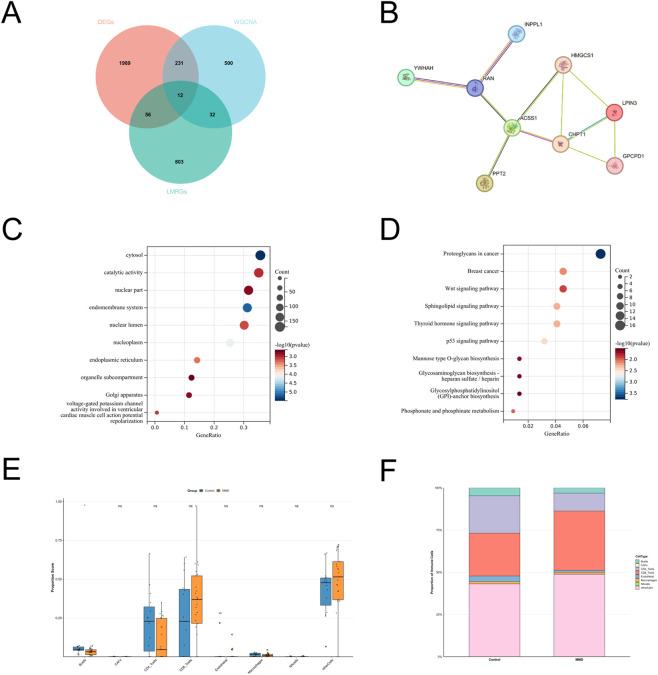
Functional enrichment analysis of DEGs and PPI network. **(A)** Venn diagram showing the key genes screened by three methods (DEGs, WGCNA, LMRGs). **(B)** PPI co-expression gene network. **(C,D)** GO and KEGG analysis of co-expressed genes. **(E,F)** Enrichment scores of eight immune infiltration cell types in all samples were calculated using the EPIC algorithm. Subsequently, we generated group comparison plots to show the correlation between the estimated percentages of immune cell infiltration in the Control group and the MMD group. Statistical tests: Wilcoxon rank-sum test (P < 0.05*; P < 0.01**; P < 0.001***; ns (no significant)).

**FIGURE 4:**
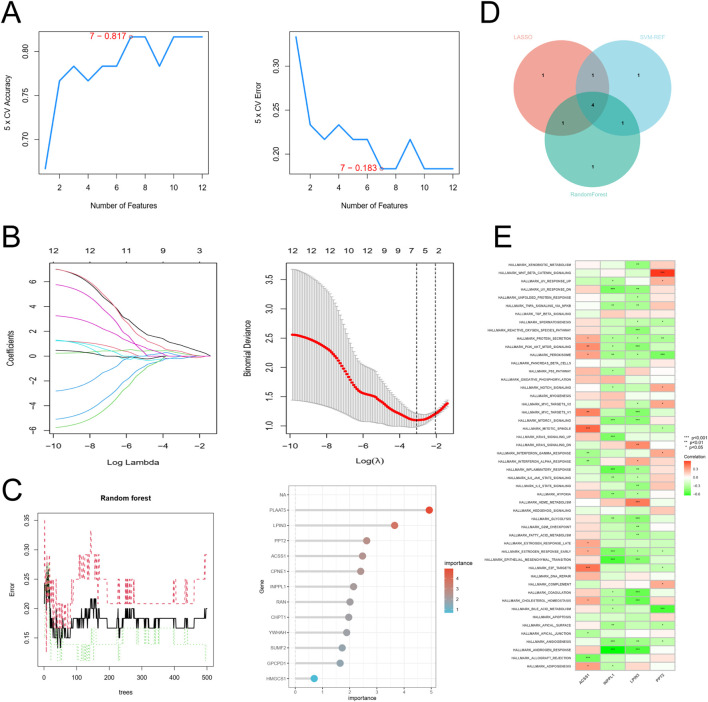
Hub gene screening through machine learning. **(A)** Feature gene screening using the SVM-RFE algorithm. **(B)** Ten-fold cross-validation performance of the tuned parameters in LASSO for feature gene screening. **(C)** Relationship between the error rate of random forest and the number of classification trees. Genes with an importance score greater than 2 were identified as feature genes by the RF model. **(D)** Venn diagram showing the hub genes screened by the three machine learning methods. **(E)** Correlation analysis of the four optimal hub genes with hallmark gene sets. Statistical test: Spearman correlation test (P < 0.05*; P < 0.01**; P < 0.001***; ns (no significant)).

Three machine learning algorithms were employed to identify feature genes, using a set of 12 significantly differentially expressed genes as input (Figure 3A): i) support vector machine-recursive feature elimination (SVM-RFE): using a linear kernel function, features were recursively eliminated via 10-fold cross-validation, resulting in the retention of seven key genes (Figure 4A); ii) least absolute shrinkage and selection operator (LASSO): a 10-fold cross-validation was performed to select the feature subset corresponding to the minimumλ(lambda) value, ultimately retaining seven key genes (Figure 4B); iii) the random forest (RF) algorithm; the model was built with ntree = 500, and features were ranked by their importance based on the Gini index. Genes with an importance value greater than 2 were selected as key genes (Figure 4C). Intersection analysis using a Venn diagram of the gene sets identified by these three methods revealed four hub genes: LPIN3, PPT2, ACSS1, and INPPL1. Subsequently, a nomogram-based predictive model was constructed using the full set of candidate genes. Receiver operating characteristic (ROC) analysis was performed using the R package “pROC” (version 1.17.0.1) to calculate the area under the curve (AUC) and evaluate diagnostic performance. The predictive model is publicly accessible. The complete R code for constructing the predictive nomogram, along with the associated data and a comprehensive usage guide, has been deposited in Supplementary Table S6. In summary, to ensure the proper application of the model, any input expression data must be processed using the identical normalization and correction pipeline applied to the training set.

### 2.5 Functional enrichment analysis and construction of protein-protein interaction network

For gene set functional enrichment analysis, we employed Gene Ontology (GO) annotations from the R package “org.Hs.eg.db” (version 3.1.0) as the background reference. Genes of interest were mapped to this background gene set, and enrichment analysis was performed using the R package “clusterProfiler” (version 3.14.3) to obtain gene set enrichment results. For KEGG pathway analysis, the background gene set of current pathway annotations was retrieved via the KEGG REST API (https://www.kegg.jp/kegg/rest/keggapi.html) and used for a similar enrichment analysis with “clusterProfiler”. Gene sets containing fewer than 5 genes or more than 5,000 genes were excluded from analysis. Statistical significance was defined as p-value <0.05 and false discovery rate (FDR) < 0.1.

The STRING database (https://cn.string-db.org/; accessed on 12 January 2026), which catalogs known and predicted protein-protein interactions (PPIs), was used to retrieve interactions among the products of metabolic genes (Figure 3B). A PPI network was constructed using a combined interaction score threshold of >0.15.

### 2.6 Assessment of the immune landscape

**FIGURE 5:**
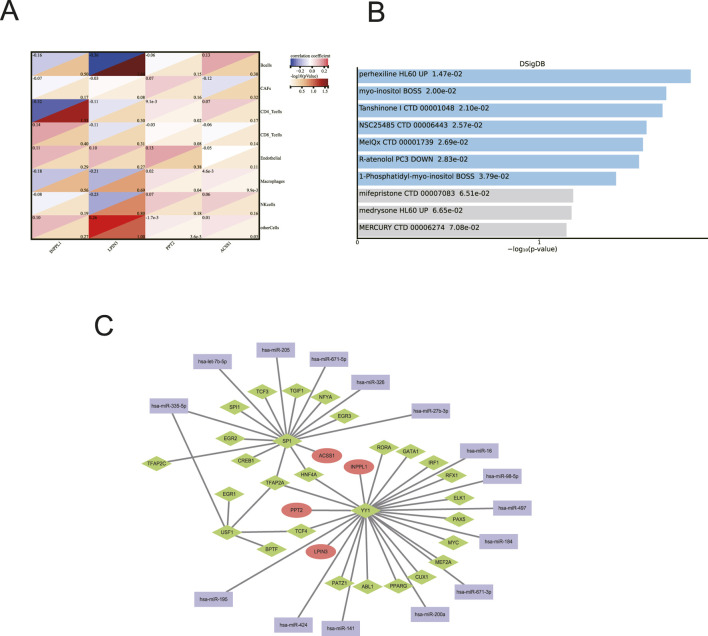
Immune infiltration analysis, potential drug prediction and TF-miRNA-mRNA network construction. **(A)** The correlation heatmap shows the results of immune cell infiltration analysis for four hub genes (LPIN3, PPT2, ACSS1, INPPL1). **(B)** Visualization of drug prediction results for hub genes using DSigDB. The blue sections represent the seven compounds with significant differences predicted. **(C)** TF-mRNA-miRNA interaction network. Red ellipses represent mRNAs, green squares represent TFs, and purple squares represent miRNAs.

EPIC (Estimating the Proportion of Immune and Cancer Cells) is a computational method designed to estimate the relative proportions of immune and cancer cells in tissue samples based on bulk gene expression data. Its core principle involves leveraging a reference matrix of cell-type-specific marker gene expression profiles and applying a linear regression framework to infer the relative abundances of distinct cell types within each sample. We utilized the IOBR R package (Zeng et al., 2021) to apply the EPIC algorithm (Racle et al., 2017) to our expression data, estimating the relative abundances of eight immune cell types across all samples. Subsequently, we generated group-comparison plots to visualize correlations between candidate genes and the estimated percentages of immune cell infiltration (Figures 3E,F), and constructed a correlation heatmap to illustrate pairwise associations among the candidate genes (Figure 5A).

### 2.7 Construction of TF-miRNA-mRNA

To identify potential regulatory interactions, we performed integrative analysis and target prediction using “NetworkAnalyst” (Zhou et al., 2019), a platform supporting network construction and visualization across multi-omics data. Three core databases were employed: miRTarBase v9.0 (Cui et al., 2025), TRRUST (Han et al., 2015), and the TF-miRNA co-regulatory network. By integrating these data, we constructed a three-layer TF-miRNA-mRNA regulatory network. The resulting network was visualized using Cytoscape software (Figure 5C).

### 2.8 Validation of independent external datasets

**FIGURE 6:**
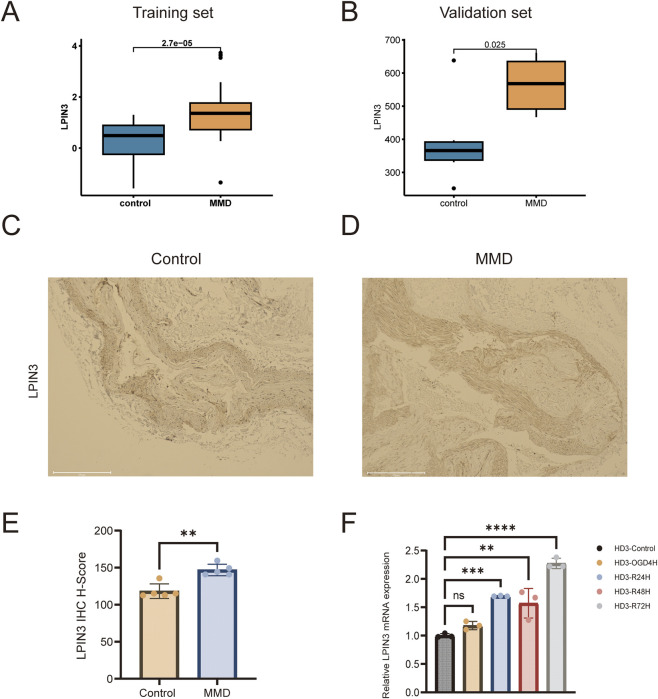
Validation of Hub Genes. **(A,B)** LPIN3 showed significant differences in the training set and validation set with a p-value of <0.05. **(C,D)** IHC staining of cerebrovascular tissues. **(E)** Histogram of IHC staining results. **(F)** Expression of LPIN3 in the HCMEC/D3 cell line under different OGD/R conditions was analyzed by qRT-PCR. Statistical test: Unpaired t-test (P < 0.05*; P < 0.01**; P < 0.001***; ns (no significant)).

To validate the research findings, the expression levels of key hub genes were independently verified in the external dataset GSE249254 (Figure 6A).

### 2.9 RT -qPCR assays for gene expression in cell lines

Immortalized human cerebral microvascular endothelial cells (HCMEC/D3; ZQ0961, ZQXZBIO), authenticated by short tandem repeat (STR) profiling, were cultured in endothelial cell-specific medium (ZM0961, ZQXZBIO). An *in vitro* oxygen-glucose deprivation/reperfusion (OGD/R) injury model was established using HCMEC/D3 cells (Ren et al., 2024; Yuan et al., 2021). For OGD induction, cells were incubated in glucose-free DMEM (Gibco) under hypoxic conditions (1% O_2_, 94% N_2_, 5% CO_2_) at 37 °C for 4 h. Subsequently, cells were returned to normoxic conditions and cultured in standard complete medium for reperfusion. This experimental paradigm was adopted based on prior studies to recapitulate the early pathophysiological microenvironment associated with MMD (Shin et al., 2024). Although no consensus exists for a cellular or animal model that recapitulates the full spectrum of MMD pathology (Ihara et al., 2022), the OGD/R model is widely used to simulate key early features such as cerebral hypoxia-ischemia, endothelial dysfunction, and vascular remodeling. For example, Shin et al. used an OGD/R model to show that impaired RNF213-mediated autophagy impairs endothelial function in MMD (Shin et al., 2024). Similarly, Ren et al. employed OGD/R on brain microvascular endothelial and smooth muscle cells to study the SDF-1/CXCR4 axis in MMD angiogenesis (Ren et al., 2024). In the present study, we employed this model solely to preliminarily validate hypoxia-induced changes in gene expression; it does not provide definitive mechanistic conclusions.

Total RNA was extracted using the RNA Rapid Purification Kit (Sangon Biotech, B511361-0100), and cDNA was synthesized via reverse transcription according to the manufacturer’s instructions (Thermo Fisher Scientific, K1622), yielding a final reaction volume of 20 μL. All qRT-PCR primer sequences are listed in Supplementary Table S3. Cycle threshold (Ct) values-the number of amplification cycles required to reach a defined fluorescence threshold-were recorded, and relative gene expression levels were calculated using the ΔΔCt method. Each qRT-PCR reaction was performed in triplicate. Gene expression levels were normalized to that of human GAPDH.

### 2.10 IHC

IHC analysis was performed on middle cerebral artery (MCA) vascular wall samples from MMD patients and non-MMD controls. The study protocol was reviewed and approved by the Ethics Committee of the First Affiliated Hospital of Xiamen University (Approval No. 2024KY273-02). Samples were fixed in 4% paraformaldehyde, paraffin-embedded, and sectioned. Briefly, sections were incubated overnight at 4 °C with a rabbit polyclonal antibody specific for LPIN3 (ABmart, PC18291S; dilution 1:200). Stained sections were imaged using a Leica light microscope, and quantitative image analysis was conducted with ImageJ software (Version 1.52).

### 2.11 Prediction of potential drugs

The Drug Signature Database (DSigDB; https://dsigdb.tanlab.org/DSigDBv1.0/, accessed on 12 January 2026) is a publicly available resource that systematically integrates drugs or chemical compounds with their target genes via curated gene sets. It currently encompasses 22,527 gene sets, representing 17,389 distinct compounds and covering 19,531 unique genes. In this study, DSigDB was used to investigate associations between the key diagnostic genes and potential therapeutic agents.

### 2.12 Statistical analysis

Statistical analyses were performed using R software (version 4.1.3). Pairwise group comparisons were performed using the Wilcoxon rank-sum test or T-test. Associations between variables were assessed using Pearson or Spearman correlation coefficients, based on tests for normality and linearity. A two-sided p-value <0.05 was considered statistically significant.

## 3 Results

### 3.1 Identification of differentially expressed genes (DEGs) and gene set enrichment analysis (GSEA)

To integrate multiple gene expression datasets and identify DEGs associated with MMD, we merged the GSE189993, GSE157628, and GSE141024 datasets using the “inSilicoMerging” R package. Batch effects were subsequently corrected and data normalized using the “RemoveBatchEffect” function (Figure 2A), yielding an integrated dataset comprising 36 MMD samples and 24 control samples. Differential expression analysis was then performed using the “limma” package, identifying 2,288 genes that met the significance thresholds of |log_2_ fold change| > 0.5 and p-value <0.05 (Figure 2B). To elucidate the biological pathways dysregulated in the integrated MMD transcriptomic profile, we conducted GSEA. Classical enrichment plots revealed strong positive enrichment of multiple immune-related gene sets, including “Allograft rejection”, “Systemic lupus erythematosus”, and “Autoimmune thyroid disease”. Notably, the “Fat digestion and absorption” pathway was also significantly enriched, suggesting a potential role for lipid metabolism in MMD pathogenesis. Ridge plot visualization of significantly enriched pathways further corroborated the pronounced upregulation of immune and inflammatory processes. These included pathways central to adaptive immunity and autoimmunity—such as “Allograft rejection”, “Graft-versus-host disease”, and “Autoimmune thyroid disease”—as well as those governing immune cell trafficking and intercellular communication, exemplified by the highly significant enrichment of “Cytokine-cytokine receptor interaction” (adjusted P-values ranging from 0.001 to 0.004). Collectively, these findings reveal a transcriptional signature of MMD characterized by robust immune-inflammatory activation, highlighting adaptive immune engagement, an autoimmune-like state, dysregulated intercellular communication, and concomitant perturbations in specific metabolic functions.

### 3.2 Identification of co-expression modules utilizing WGCNA

**FIGURE 7:**
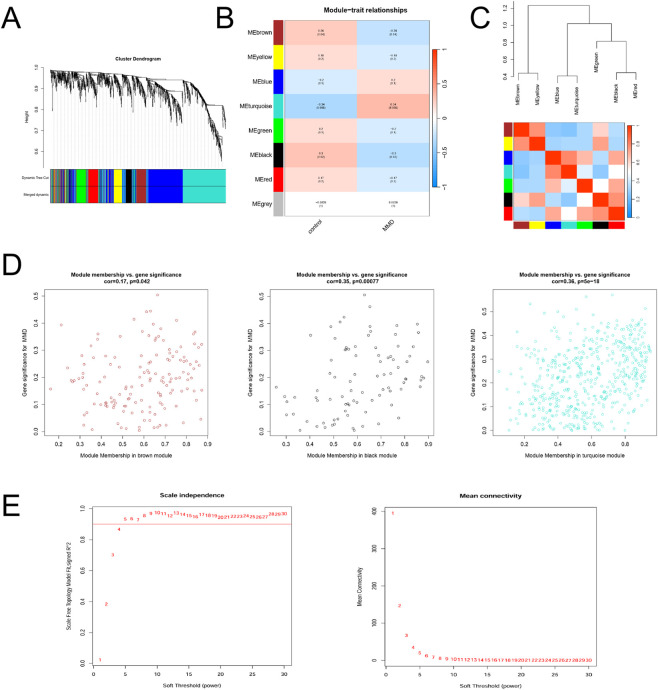
Construction of WGCNA co-expression network. **(A)** Tree-ring plot of gene clusters with median absolute deviations in the top 25%. Each branch in the figure represents a gene, and each color below represents a co-expression module. **(B)** Heatmap of module-trait correlations. Red indicates positive correlation, and blue indicates negative correlation. **(C)** Heatmap of correlations among module characteristic genes. Red indicates high correlation, and blue indicates the opposite result. **(D)** Correlations between the three modules (turquoise, black, brown) and MMD. **(E)** A soft-thresholding power (β) of 4 was selected to ensure scale-free topology, achieving a model fit index (R^2^) > 0.9.

WGCNA was employed to analyze the gene expression profiles of the combined MMD set. This analysis involved constructing a gene co-expression network and identifying co-expression modules using the WGCNA package in R. The dataset comprised 24 control samples and 36 MMD samples, which were subjected to hierarchical clustering. Samples exhibiting pronounced outliers were excluded based on predefined thresholds. Subsequently, eight co-expression modules were identified using a combination of gene dendrogram-based clustering and the dynamic tree-cutting algorithm, with a minimum module size of 50 genes (Figures 7A,B). A scale-free network was constructed using a soft-thresholding power of β = 4 (scale-free R^2^ = 0.9), ensuring high network connectivity (Figure 7E). Among all modules, the Turquoise module showed the strongest correlation with MMD status (r = 0.34, P = 0.008). Gene significance for MMD-associated genes was statistically significant in the Brown (cor = 0.17, P = 0.042), Black (cor = 0.35, P = 0.00077), and Turquoise (cor = 0.36, P = 5e-18) modules (Figure 7D). Finally, overlapping genes from these three modules were combined and used for the screening of hub genes.

### 3.3 Functional enrichment analysis and PPI network establishment based on LMDEGs

Lipid metabolism-disease-associated differentially expressed genes (LMDEGs) were defined by intersecting the DEGs with genes from the WGCNA-derived modules and the lipid metabolism-related gene (LMRG) set. Genes meeting the thresholds of |log_2_FC| > 0.5 and p-value <0.05 were retained as LMDEGs. Heatmap visualization of these results was performed using the R package “pheatmap”. Subsequently, 12 LMDEGs were selected for Venn diagram representation (Figure 3A). GO enrichment analysis revealed that these LMDEGs were significantly enriched in biological processes and cellular components including “cytosol”, “catalytic activity”, “nuclear part”, “endomembrane system”, and “nuclear lumen” (Figure 3C). KEGG pathway enrichment analysis highlighted prominent associations with “Proteoglycans in cancer”, “Breast cancer”, “Wnt signaling pathway”, and “p53 signaling pathway” (Figure 3D). Collectively, these findings suggest that the pathogenesis of MMD may be closely linked to dysregulation of cancer-related signaling pathways, aberrant cell cycle regulation, and disturbances in the synthesis and metabolism of extracellular matrix components. To explore functional interactions, the 12 most significantly dysregulated LMDEGs from the training set were used to construct a PPI network via the STRING database, applying a minimum required interaction score of 0.15. Following stringent topological filtering, nine hub genes exhibiting strong interconnectivity were retained for downstream analysis (Figure 3B).

### 3.4 Selection and validation of signature genes and construction of diagnostic models

**FIGURE 8:**
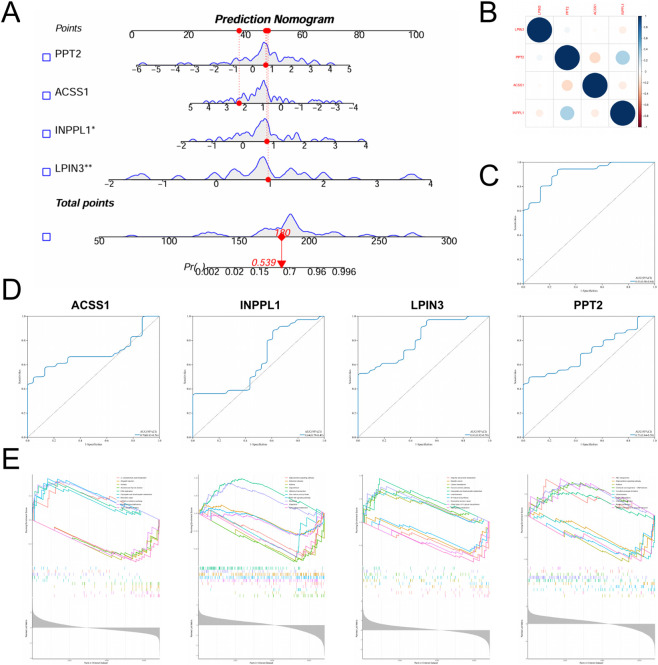
Diagnostic value of hub genes and GSEA analysis. **(A,C)** Diagnostic nomograms were constructed using the selected hub genes, followed by ROC analysis of the model. **(B)** Correlation analysis among hub genes. **(D)** ROC curves of hub genes, with the area under the curve (AUC) values indicated in the legend, representing the diagnostic performance of each gene in differentiating MMD from control samples. **(E)** GSEA analysis of hub genes.

In this study, three machine learning algorithms were employed to identify feature genes. A Venn diagram illustrating the intersection of gene sets identified by these three methods revealed four overlapping hub genes: “LPIN3”, “PPT2”, “ACSS1”, and “INPPL1” (Figure 4D). Pathway enrichment scoring of the expression profiles of these hub genes was performed using single-sample gene set enrichment analysis (ssGSEA), with the “h.all.v2025.1. Hs.symbols” gene set. The resulting pathway scores were visualized as a heatmap (Figure 4E). Notably, the expression of these hub genes strongly correlated with several hallmark pathways. Subsequently, a diagnostic nomogram for MMD was developed using the four identified hub genes (LPIN3, PPT2, ACSS1, and INPPL1) (Figure 8A). The expression correlation analysis among the four identified core genes (LPIN3, PPT2, ACSS1, and INPPL1) is presented in Figure 8B. The model demonstrated robust diagnostic performance, achieving an area under the receiver operating characteristic curve (AUC) of 0.91 (Figure 8C). Among the individual features, LPIN3 alone yielded an AUC of 0.81 (Figure 8D). Performance metrics from the independent validation set (GSE249254, 6 MMD patients and 6 controls) demonstrated the diagnostic potential of the identified biomarker. Specifically, LPIN3 alone achieved an AUC of 0.89 (95% CI: 0.66–1.00). Data for the other hub genes have been provided in Supplementary Figure D. GSEA was further conducted to elucidate signaling pathways associated with the hub genes (Figure 8E). GSEA of LPIN3 revealed significant positive enrichment in the “Glyoxylate and dicarboxylate metabolism” and “Nucleotide excision repair” pathways. These findings suggest that LPIN3 may contribute to MMD pathogenesis by promoting genomic stability and metabolic homeostasis-potentially through activation of DNA damage repair mechanisms and energy metabolism-related pathways.

### 3.5 Analysis of immune cell infiltration based on the DEGs (EPIC)

To further investigate differences in immune infiltration between MMD and control samples, we performed a comprehensive analysis using IOBR (Zeng et al., 2021), a computational toolkit for immuno-oncology research. Specifically, using the R package IOBR, we applied the EPIC algorithm (Mootha et al., 2003; Racle et al., 2017) to quantify immune cell abundances based on our gene expression profiles, yielding correlation scores for eight major immune cell types across the training set samples (Figures 3E,F). While CD8^+^ T cells, CD4^+^ T cells, and B cells showed relatively high overall infiltration levels, B-cell abundance was significantly lower in the MMD group compared to controls. Moreover, B-cell infiltration showed strong and statistically significant correlations with the expression levels of several characteristic genes-including LPIN3, PPT2, and INPPL1-which are implicated in the pathogenesis and progression of MMD (Figure 5A). In addition, CD8^+^ T cells and endothelial cells demonstrated robust correlations with hub genes, suggesting that dysregulated lipid metabolism may contribute to disease progression by modulating immune responses and participating in vascular remodeling.

### 3.6 TF-miRNA-mRNA interaction networks and drug prediction

Using the NetworkAnalyst platform, we predicted microRNAs (miRNAs) and transcription factors (TFs) targeting the four hub genes (LPIN3, PPT2, ACSS1, and INPPL1). A regulatory network was constructed based on interaction data retrieved from its integrated databases and visualized using Cytoscape (Figure 5C). Notably, three transcription factors (TFs)-YY1, SP1, and USF1-were identified as the most strongly connected regulators to the hub genes within this TF-miRNA-mRNA network. Detailed interaction information is provided in Supplementary Table S4. To explore potential therapeutic agents, drug set enrichment analysis was performed for the four hub genes using Enrichr with the DSigDB library (accessed 12 January 2026), and the significant drug-gene associations are summarized in a bar plot (Figure 5B; Supplementary Table S5).

### 3.7 Validation of independent external datasets and experimental

To validate the expression of the candidate hub genes, we first analyzed the independent GEO validation dataset. LPIN3 was the only hub gene that exhibited significant differential expression in both the training set and the independent validation set (Figures 6A, B; Supplementary Figure SA). To further investigate the expression patterns of hub genes in MMD, we established a hypoxia model using the HCMEC/D3 cell line. qRT-PCR analysis revealed that hypoxia significantly upregulated LPIN3, PPT2, and INPPL1 mRNA levels, while downregulating ACSS1(Figure 6F; Supplementary Figure SB). As LPIN3 was the only hub gene validated in both independent datasets and the functional model, we focused subsequent IHC validation on LPIN3 protein expression in MCA tissues from MMD patients and controls. In parallel, IHC confirmed significantly elevated LPIN3 protein levels in MMD samples compared to controls (Figures 6C–E). Together, these multi-level validation data establish LPIN3 as a promising biomarker candidate for MMD.

## 4 Discussion

This study integrates bioinformatic analyses to provide novel molecular insights into the immunometabolic crosstalk underlying MMD, and proposes a promising panel of diagnostic biomarkers. LPIN3, PPT2, ACSS1, and INPPL1 were identified as core hub genes. Notably, the study reveals LPIN3 as a previously unrecognized biomarker positively associated with MMD risk, highlighting a critical interplay between dysregulated lipid metabolism and immune dysfunction in the pathogenesis of MMD.

Typical histopathological features of occluded arteries in MMD include intimal fibrocellular thickening and the presence of proliferating smooth muscle cells within the intima. Previous studies have primarily focused on genetic susceptibility and immune-mediated vascular changes in MMD (Asselman et al., 2022; Ye et al., 2023). However, dysregulation of specific lipoprotein subclasses, notably in lipid metabolism, is increasingly recognized as a contributor to both disease onset and clinical severity (Mineharu et al., 2025; Patel et al., 2026). Clinical studies have shown a high prevalence of dyslipidemia, primarily hypertriglyceridemia and low high-density lipoprotein cholesterol (HDL-C), among MMD patients in China (Wang et al., 2024). Moreover, patients with the ischemic subtype exhibit significantly higher total cholesterol (TC), triglyceride (TG), and low-density lipoprotein cholesterol (LDL-C) levels than those with the hemorrhagic subtype. Accumulating evidence suggests that lipid metabolism disorders may elevate the risk of ischemic stroke in MMD patients (Yu et al., 2022). Furthermore, a prospective study of postoperative MMD patients identified non-alcoholic fatty liver disease (NAFLD) as an independent risk factor for stroke following revascularization surgery and subsequent neurological deterioration (Zhang et al., 2024). These findings suggest that lipid-lowering interventions and restoration of lipid homeostasis may help prevent stroke in MMD. Additionally, HDL dysfunction, particularly impaired antioxidant capacity, has been linked to MMD-related vascular pathology (Zhang J. et al., 2025; Ge et al., 2020). Therefore, integrating lipid metabolism dysregulation with immune-mediated vascular mechanisms and other pathobiological factors may enhance our mechanistic understanding of MMD and facilitate the identification of more robust and clinically relevant biomarkers.

Our findings significantly advance the understanding of the molecular underpinnings of MMD by providing a multifaceted view of its transcriptomic landscape. First, GSEA revealed robust positive enrichment of multiple immune-related pathways—including “Allograft rejection”, “Systemic lupus erythematosus,” and “Autoimmune thyroid disease”. These results are consistent with growing evidence implicating chronic inflammation and autoimmune-like responses in MMD pathogenesis (Ge et al., 2024; Kanamori et al., 2021). At the transcriptomic level, our data corroborate these observations from a systems perspective, indicating broad activation of the adaptive immune system. Notably, the enriched pathways also included “Fat digestion and absorption”, suggesting a potential role for lipid metabolism in MMD progression. This finding resonates with recent metabolomic studies: for instance, He et al. reported dysregulated diacylglycerol and triacylglycerol metabolism in MMD arterial tissue (He et al., 2023), and Chen et al. identified lipoprotein(a) as a risk factor for MMD (Chen et al., 2024). Collectively, our integrative analysis links immune activation with specific metabolic perturbations in MMD. This suggests that lipid metabolic abnormalities are not mere epiphenomena but active contributors to pathogenesis, which may sustain and amplify a pro-inflammatory state, thereby driving disease progression.

The identification of four hub genes—LPIN3, PPT2, ACSS1, and INPPL1—through a rigorous machine learning pipeline represents a key contribution. A diagnostic model constructed using these genes achieved an impressive AUC of 0.91, underscoring their collective predictive utility. Of particular interest is LPIN3, a gene implicated in lipid metabolism. LPIN3 is essential for phosphatidic acid phosphatase (PAP) activity, catalyzing the conversion of phosphatidic acid to diacylglycerol, a key step in lipid synthesis and signaling (Csaki et al., 2014; Zhang X. et al., 2025). Our experimental validation confirmed significant upregulation of LPIN3 protein in hypoxic endothelial cells and in patient-derived vascular samples. The expression profile of LPIN3 in other cerebrovascular diseases, such as intracranial aneurysm (IA) and intracranial atherosclerotic stenosis (ICAS), remains insufficiently investigated. This knowledge gap suggests that LPIN3 may possess disease-specific characteristics, supporting its potential as a biomarker specific to MMD. This finding appears distinct from prior transcriptomic studies that predominantly emphasized immune-related genes; however, it strongly supports the growing recognition of metabolic dysregulation in cerebrovascular disease (He et al., 2025b; Yu et al., 2022). For instance, a prospective case-control study by Ge et al. reported an inverse association between elevated high-density lipoprotein (HDL) cholesterol levels and MMD risk (Ge et al., 2020), providing indirect evidence for the involvement of the lipid pathway in which LPIN3 operates. Supporting this, animal studies show that lipin-1/lipin-3 double-knockout mice have severely reduced adipose PAP activity, indicating functional redundancy among lipin isoforms in maintaining lipid homeostasis (Csaki et al., 2014). Moreover, our hub genes—especially LPIN3—showed significant correlations with altered B-cell and T-cell infiltration levels, further reinforcing the emerging hypothesis of metabolic-immune crosstalk within the vascular wall (Dekkers et al., 2023; Robinson et al., 2022), a concept gaining increasing traction across diverse vascular pathologies. Our analysis of immune cell infiltration, performed using the EPIC algorithm, revealed a marked reduction in B cells within the MMD samples. This finding provides novel insights that enhance the current understanding of the disease’s immunopathology.

In the peripheral immunopathology of MMD, T-cell dynamics have been well-characterized, primarily as disturbances in T-cell subsets, including decreased effector T cells and increased regulatory T cells, and along with elevated B-cell and monocyte counts (Ge et al., 2024; Demirag et al., 2025). However, this peripheral immune profile does not directly mirror the cellular landscape within the MMD vascular wall. Our analysis of immune cell infiltration in vascular wall tissue, using the EPIC algorithm, demonstrated a marked reduction in B cells in MMD samples. This finding provides a novel and complementary perspective on the prevailing understanding of MMD immunopathology. Through scRNA-seq of superficial temporal artery specimens from MMD patients, He et al. (2025b) identified natural killer T (NKT) cells as playing a critical role in arterial intimal thickening by promoting the proliferation, migration, and cytoskeletal expansion of endothelial cells and smooth muscle cells. The strong correlations we observed between LPIN3 and two types of immune cells—macrophages and NK cells—are consistent with the findings reported by He et al. LPIN3 may contribute to the progression of MMD by mediating the infiltration of NK cells. The role of B cells in MMD pathogenesis, however, remains unclear, partly due to limitations in sample availability. Our observation of reduced B-cell infiltration, along with its significant correlation with key hub gene expression (e.g., LPIN3, INPPL1, Figure 5A). LPIN3-mediated disruption of lipid metabolism may remodel the landscape of B-cell infiltration in the vascular wall, representing a key mechanism contributing to the progression of MMD. The recruitment or survival of B cells within diseased vessels is likely governed by more complex regulatory networks, warranting further experimental investigation.

We acknowledge several limitations of our study. First, the analysis is based on bulk transcriptomic data, and the scarcity of clinical samples limited our ability to standardize the vascular tissue sources in the control group. Although we performed data integration and batch correction, residual confounding cannot be ruled out. Second, it is important to note that the sample size of the independent validation set was limited (n = 12). Although the hub gene LPIN3 maintained a high diagnostic performance, the external validation of the model may still be associated with limited reliability and suboptimal sensitivity. Therefore, further validation with an expanded local sample cohort is warranted in future studies. Third, the precise cellular mechanisms of LPIN3 and other key genes remain to be elucidated. Although we validated LPIN3 *in vitro* and in patient samples, the OGD/R model cannot fully recapitulate the complex pathological microenvironment of MMD, and the absence of a robust animal model precludes recreation of the disease’s full vascular context; consequently, the precise functional contributions of these genes remain incompletely defined. Future studies should investigate the functional links between these key genes and the established genetic factor RNF213 to identify potential convergent pathways. Future studies should incorporate samples from patients with IA and ICAS to further validate the diagnostic specificity of LPIN3 for MMD. Additionally, evaluating the therapeutic potential of LPIN3 as a drug target and verifying its diagnostic value in larger, independent patient cohorts will be crucial for advancing clinical management strategies for MMD.

## 5 Conclusion

In summary, through integrated bioinformatics analysis coupled with experimental validation, we identified LPIN3 as a key biomarker associated with immune infiltration and lipid metabolism in MMD.

## Data Availability

The datasets presented in this study can be found in online repositories. The names of the repository/repositories and accession number(s) can be found in the article/Supplementary Material.
