# Supplementary figures and images for: LPIN3 emerges as a diagnostic biomarker in Moyamoya disease revealing immune-lipid metabolic crosstalk
Source: Front Genet. 2026 Jun 9;17:1853818. doi: 10.3389/fgene.2026.1853818 (PMC13286443; doi:10.3389/fgene.2026.1853818)

**A**


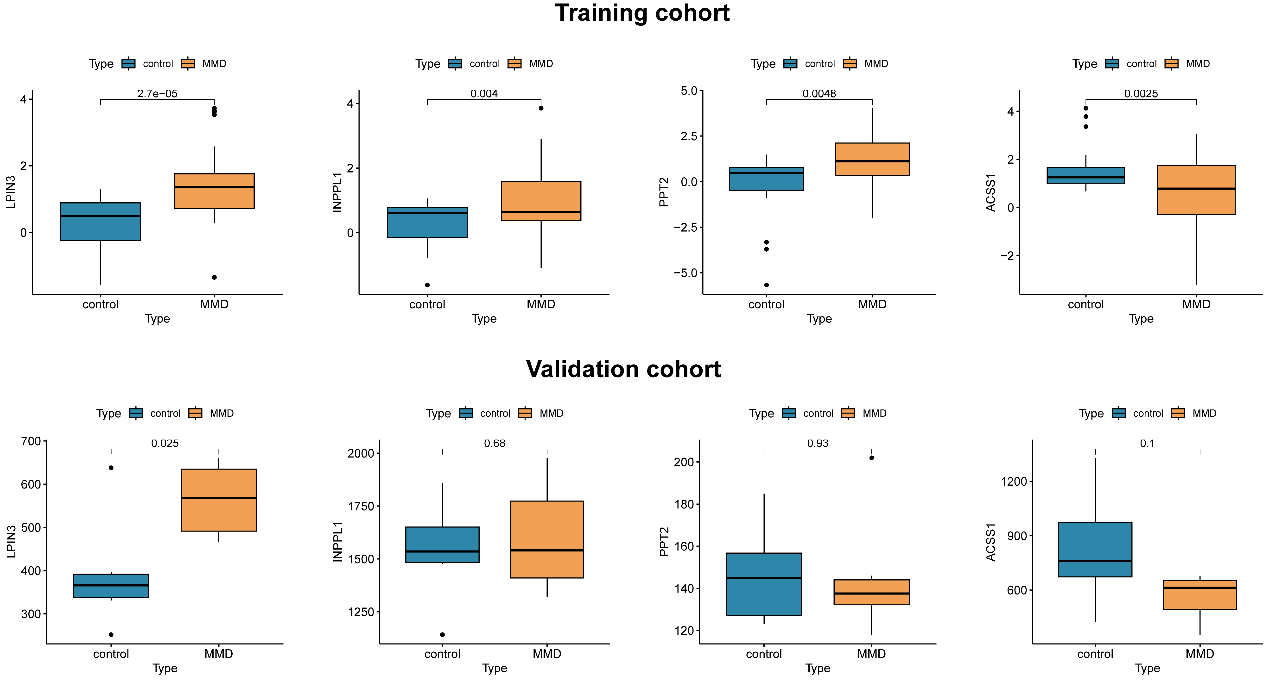


**B**


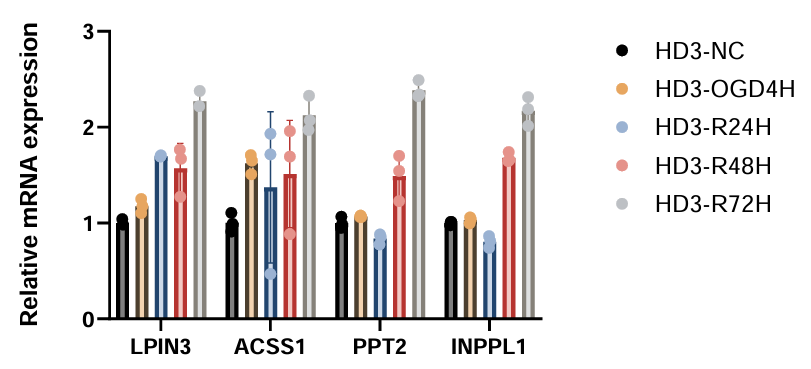


**C**


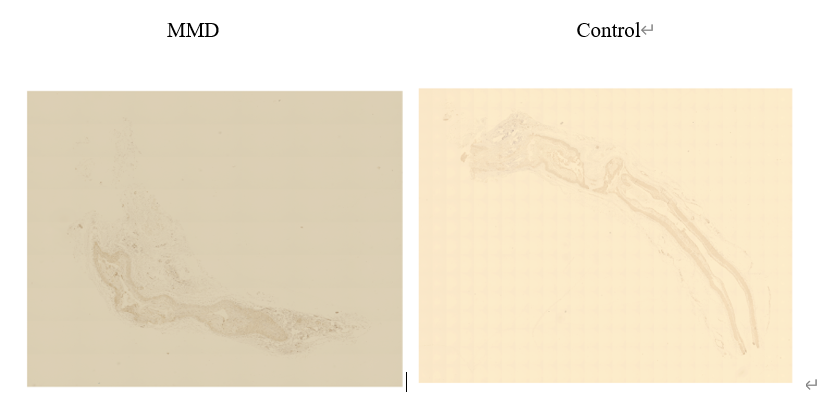


D


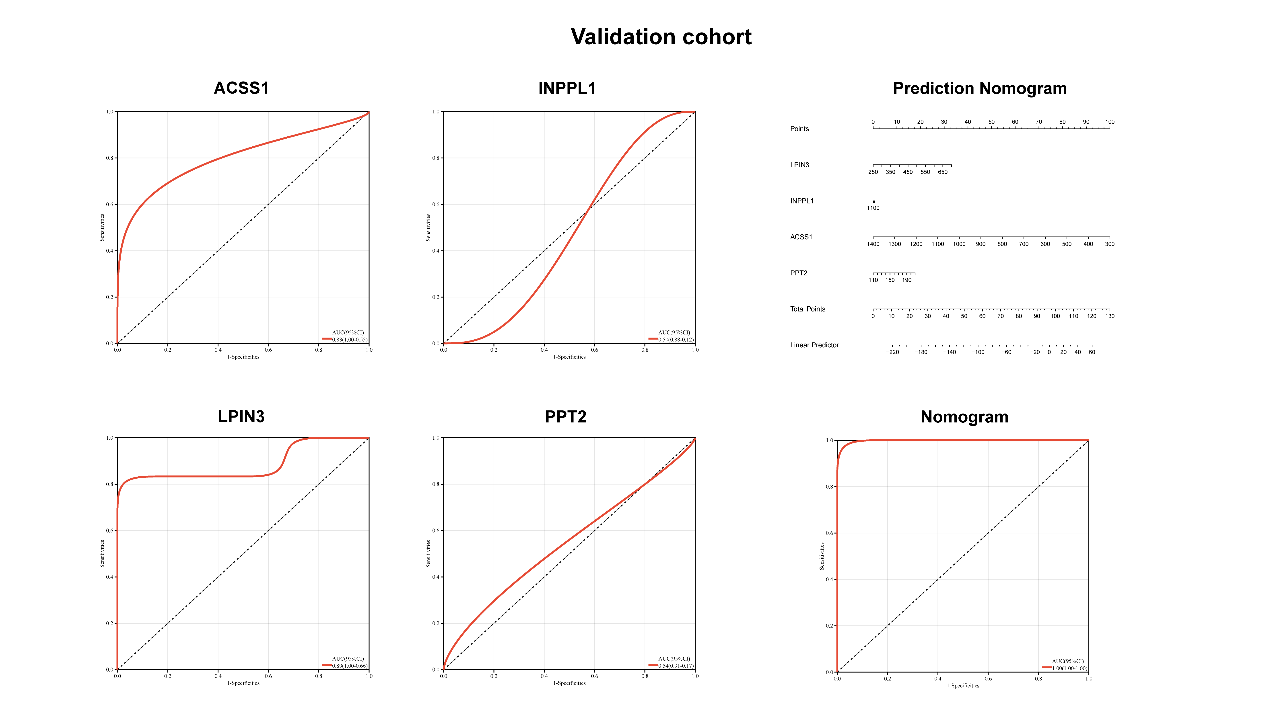

Supplement: Supplementary file 1 [file Supplementaryfile1.docx]
